# ASPM-associated stem cell proliferation is involved in malignant progression of gliomas and constitutes an attractive therapeutic target

**DOI:** 10.1186/1475-2867-10-1

**Published:** 2010-01-11

**Authors:** Sandra-Nadia Ngwabyt Bikeye, Carole Colin, Yannick Marie, Raphaël Vampouille, Philippe Ravassard, Audrey Rousseau, Blandine Boisselier, Ahmed Idbaih, Charles Félix Calvo, Pascal Leuraud, Myriam Lassalle, Soufiane El Hallani, Jean-Yves Delattre, Marc Sanson

**Affiliations:** 1 UMR975 INSERM-UPMC, GH Pitié- Salpêtrière, Paris; 2 UMR911-CRO2, Faculté de Médecine Timone, Université de la Méditerranée, Marseille; 3 Service de Neurologie Mazarin, GH Pitié- Salpêtrière, APHP, Paris; 4 Biotechnology & Biotherapy laboratory, Centre de Recherche de l'Institut du Cerveau et de la Moelle, CNRS UMR9225, INSERM UMRS 975 Université Pierre et Marie Curie, Hôpital de la Salpêtrière, Paris; 5 Service de Neuropathologie Raymond Escourolle, GH Pitié- Salpêtrière, Paris; 6 XenTech, Evry, France

## Abstract

**Background:**

ASPM (*Abnormal Spindle-like Microcephaly associated*) over-expression was recently implicated in the development of malignant gliomas.

**Results:**

To better characterize the involvement of ASPM in gliomas, we investigated the mRNA expression in 175 samples, including 8 WHO Grade II, 75 WHO Grade III and 92 WHO Grade IV tumors. *Aspm *expression was strongly correlated with tumor grade and increased at recurrence when compared to the initial lesion, whatever the initial grade of the primary tumor. ASPM expression also increased over serial passages in gliomaspheres *in vitro *and in mouse xenografts *in vivo*. Lentivirus-mediated shRNA silencing of ASPM resulted in dramatic proliferation arrest and cell death in two different gliomasphere models.

**Conclusion:**

These data suggest that ASPM is involved in the malignant progression of gliomas, possibly through expansion of a cancer stem cell compartment, and is an attractive therapeutic target in glioblastoma multiforme.

## Introduction

Gliomas are the most common type of primary tumors in the central nervous system (CNS), and their usual fate is to recur, either as a similar or more frequently as a higher grade lesion, explaining their grim prognosis. The cellular origin of gliomas remains unknown, but accumulating data suggest that transformed, stem-like precursors (CSC) may represent the cells that drive their growth [1,2].

The product *of ASPM *gene (*Abnormal Spindle-like Microcephaly associated*) localizes to centrosomes, spindle poles, and the midbody [3]. By promoting neuroblast proliferation and driving the orientation of mitotic cleavage, it allows for symmetric, proliferative division of neuroepithelial cells during brain development, and consequently brain surface expansion [3]. Mutations within this gene produce primary microcephaly [4]. In addition to its role in embryonic development, ASPM is highly expressed in many tumor cell lines [5], suggesting an important involvement of this gene during tumorigenesis. Recent evidence suggests that ASPM is also overexpressed in malignant gliomas and that its knockdown inhibits tumor proliferation [6,7].

In this study, we report that ASPM expression is correlated with tumor grade and increases at recurrence or after consecutive passages *in vitro *and *in vivo*. Furthermore, we found that shRNA-mediated inhibition of ASPM results in dramatic growth inhibition and extensive cell death in gliomaspheres.

## Materials and methods

### Patients and tissue samples

Tumor samples frozen in liquid nitrogen were obtained from patients who underwent surgery for a glioma at our institution and gave informed consent for molecular research studies. Histological diagnoses were made according to the World Health Organization (WHO). BAC-array-based Comparative Genomic Hybridization analysis (CGH-array) was performed as previously described [8]. Non-tumoral (NT) control brain (cerebellum and white matter) was obtained from injured or epileptic patients (cryptogenetic epilepsy) who underwent surgery.

### Total RNA isolation, reverse transcription (RT) and quantitative real-time PCR (qPCR)

Total RNA extraction was performed using RNAble blue solution (Eurobio, France) according to the manufacturer's instructions. Total RNA was systematically analyzed on both a spectrophotometer and an Agilent 2100 bioanalyzer (Agilent Technologies, FRANCE). cDNA was prepared from 1 μg RNA using a combination of random primers (48190-011; Invitrogen, France) and MMLV reverse transcriptase (28025-013; Invitrogen, France).

Gene expression was quantified by Syber Green real-time qPCR analysis (Absolute solute Syber Green Rox Mix, Abgene, Paris, France). Real-time qPCR cycles were as follows: initial denaturation at 95°C for 15 minutes, 95°C for 30 seconds and annealing at 60°C for 1 min. Cycles were repeated 40 times. Primers were as follows: ASPM, Quantitect Primers Assay (ref: QT00020209 Qiagen). The amount of cDNA was normalized to the amount of the *ALAS *housekeeping gene (Human Erythroid 5-Aminolevulinate Synthase) using primers F: 5'-TGCAGTCCTCAGCGCAGTCT-3' and R: 5'-TGGCCCCAACTTCCATCAT-3'. The 2^-ΔΔCT ^equation was applied to calculate the relative copy number of tumor samples *versus *non-neoplastic tissues.

### Cell Isolation and culture

Briefly, tumors were immediately collected after surgery in HBSS (14175, Invitrogen) and dissected after removal of gross necrosis, subjected to a trypsin digestion (5 mg/mL) for 10 min at 37°C, and then trituration and filtering. The cell suspension obtained was then plated at a density of 5.10^5 ^cells/75 cm^2 ^flask (Corning, FRANCE) in 10 mL of DMEM/F12 medium supplemented with Insulin (5 mg/mL), Penicillin - Streptomycin (50 mg/mL) and growth factor (bFGF; 20 ng/mL), epidermal growth factor (EGF; 20 ng/mL) and B27 (1/50) as a stem cell-permissive medium and maintained in a 5.0% CO2/95% O2 atmosphere with 100% humidity. Cultures were fed every three days by changing half of the medium. Primary gliomaspheres were obtained after 5-10 days.

The U87 and U251 glioblastoma cell lines and the HEK293T kidney cell line were purchased from ATCC (American Type Culture Collection, USA), grown on DMEM 10% FCS- 1% Penicillin-Streptomycin, and used as controls for *in vitro *experiments.

### LV-shRNA transduction and viability Fluorescence-activated cell sorter analysis of Gliomaspheres

We used pGIPZ (RHS4287, Biovalley FRANCE) lentiviral vectors expressing a short hairpin RNA (LV- shRNAmir, Biovalley, FRANCE) for ASPM silencing (sequence: TGCTGTTGACAGTGAGCGCGGCATTGGCGTGCTTATTTAATAGTGAAGCCACAGATGTATTAAATAAGCACGCCAATGCCTTGCCTACTGCCTCGGA or a scrambled non-targeted shRNA (RHS 4346; Biovalley FRANCE.) as a control.

Lentiviral vector stocks were produced by transient transfection of HEK293T cells with the p8.91 encapsidation plasmid [9], the VSV glycoprotein-G-encoding pHCMV-G plasmid [10], and the lentiviral recombinant vector as previously described [11]. Supernatants were treated with DNAse I (Roche Diagnostic) prior to ultracentrifugation, and the resulting pellet was resuspended in Phosphate Buffered Saline, separated into aliquots and frozen at -80°C until use. The transduction efficiency of each vector stock was determined by FACS analysis as previously described [12].

Gliomasphere cultures were mechanically disaggregated in order to increase the efficiency and uniformity of infection and then transduced at 3-5.10^6 ^cell/mL with LV-sh-miR of ASPM and non-targeted shRNA at a multiplicity of infection of 2.5 (MOI: number of transducing units per cell) supplemented with DEAE dextran (20 μg/mL). After expansion of the culture, GFP-positive viable cells were separated by cell sorting and flow cytometry FACS (FACS Aria™ PQ1300010, BD Biosciences) to ensure knockdown efficiency. After 7 days, ASPM RNA expression was measured in glioma stem cells infected with lentiviruses by real-time qPCR.

### Proliferation and cell death quantification

Proliferation was assessed by EDU incorporation (Click iT™ EdU Flow Cytometry Assay Kits, A10202 Invitrogen), and cell death by 7-AAD^+^cell staining. The percentage of labeled cells was quantified by cell sorting on flow cytometry FACS (FACS Aria™ PQ1300010, BD Biosciences).

### Subcutaneous Xenograft formation assay

2 to 5 × 10^5 ^cells suspended in 100 μl of HBSS medium were injected subcutaneously into *nude *mice. Before xenograft establishment, tumors were passed four times and genomic stability was checked by CGH array analysis.

### ASPM Immunofluorescence

To detect the endogenous ASPM protein, cultured cells were fixed for 15 min in 4% paraformaldehyde, washed twice in PBS, treated for 30 min with 0.1 Triton X-100 -5% FCS, and incubated for 30 minutes at room temperature with Rabbit-anti-ASPM (IHC Antibody Affinity purified Cat IHC_00058 BETHYL, dilution 1:50) in 0.1 Triton X-100 -1% FCS. After two washes in 0.1 Triton X-100 -1% FCS, samples were incubated for 30 minutes at room temperature with goat anti-rabbit IgG-FITC (dilution 1:1000) in 0.1 Triton X-100 -1% FCS. After two washes, samples were stained with Hoechst solution and rinsed with PBS. Samples were mounted with Fluoromount^® ^(0100-01, SouthernBiotech, France). Images were captured with a Zeiss microscope (Axiophoto) equipped with a cooled CCD camera (Cool SNAP HQ, Photometrics, Tucson, AZ, USA and analyzed by IPLab software).

### Protein extraction and ASPM Western blot

Total protein, obtained by lysing tumor tissue or cells with RIPA 1× buffer (SDS 20%, NP-40 20%, Deoxycholate acid 10%, Tris-HCl 2 M pH: 8, NaCl 5 M for 10× buffer) was quantified using a Pierce BCA™ Protein Assay Kit (ref 23225 Pierce, USA), separated on Nu-Page 3-8% Tris-acetate polyacrylamide gels (Invitrogen), and electro-transferred to nitrocellulose membranes (Bio-Rad Laboratories, Inc.) in 25 mM Tris, 192 mM glycine, 20% ethanol, and 0.01% SDS for 2.5 - 3 hours at 80-150 volts. After blocking in 5% milk solution, the membranes were probed with anti- ASPM (K-17 sc-48883) and anti- GAPDH (V-18 sc-20357) both from Santa Cruz Biotechnology, Inc., U.S.A. Antibodies were diluted 1:500 for ASPM and 1:700 for GAPDH in TBST (50 mM Tris Base, 150 mM NaCl, 0.1% (v/v) Tween-20) containing 5% milk powder. After washing in TBST, the membrane was incubated with appropriate horseradish peroxidase (HRP)-conjugated secondary antibodies diluted 1:5000 in TBST. The immunoreactive signals were detected using an ECL reagent Advance Western Blotting Detection Kit (GE Healthcare) and an Image Reader *LAS-1000 Pro ver. 2 *(KODAK PHOTO FILM 4000 mm, FRANCE).

### Statistical analysis

The χ^2 ^test was used to compare qualitative variable distribution, and the Mann-Whitney test was used for continuous variables. A two-sided p-value < 0.05 was considered to be significant.

## Results

### 1. ASPM expression increases with glioma grade

**Figure 1:**
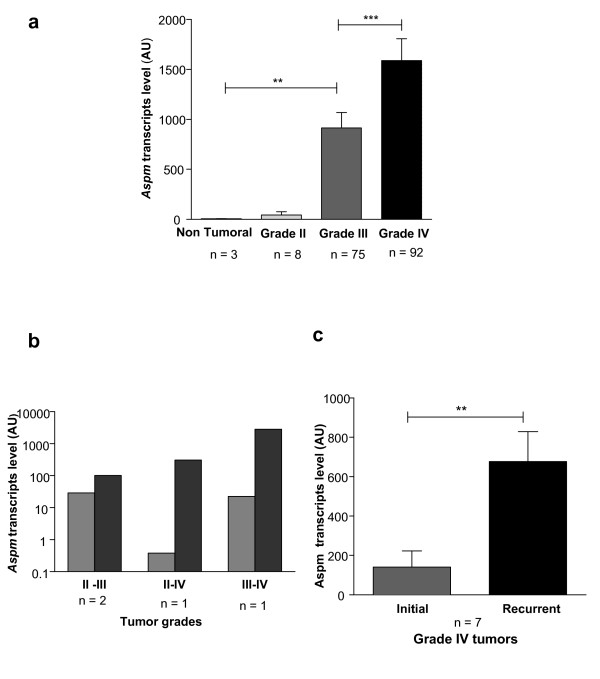
***Aspm *expression correlates with glioma grade and increases at recurrence**. **(a) ***Aspm *is up-regulated in gliomas compared to non-tumor brain and increases with the tumor grade;**(b) ASPM **expression increases between the initial tumor and recurrence at a higher grade; **(c) ASPM **expression also increases at recurrence when the initial tumor is already a Grade IV GBM. The scale bar represents the standard error of the mean (SEM).

We measured ASPM expression via qRT-PCR in 175 gliomas (8 Grade II, 75 Grade III and 92 Grade IV) and in three non-neoplastic brain tissues (NT). ASPM expression level increased between NT tissue and Grade II gliomas (p < 0.05), between Grade II and Grade III (p < 0.005), and between Grade III and Grade IV (p = 0.0001). ASPM expression did not differ between astrocytic (2 AII, 36 AIII), oligodendroglial (1 OII, 13 OIII) or mixed glioma samples (5 OAII, 26 OA III) (Figure 1a).

Preliminary results with western blot analysis showed that ASPM was also expressed at the protein level in gliomas and overexpressed in Grade IV gliomas as compared to Grades III and II (additional file 1, Figure S1).

### 2. ASPM is upregulated in recurrent gliomas relative to the initial tumor

ASPM mRNA expression was measured in 11 recurrent tumors and compared to the initial tumor (3 Grade II, 1 Grade III and 7 Grade IV). As shown in Figure 1b, increased malignancy at recurrence was identified in all 4 non-glioblastoma patients with concomitant increase of ASPM expression in all of them. In the 7 patients whose initial tumor was a GBM (Figure 1c), ASPM expression was 3.5-fold higher in the recurrent tumor although the grade did not change (p < 0.005).

### 3. ASPM expression increases with the number of passages in gliomaspheres and in xenografted tumors

Because ASPM expression increases with the recurrence of glioma, and because ASPM is involved in tumor stem cells, we investigated ASPM expression over time *in vitro *in spheroid cultures and *in vivo *on xenografts

**Figure 2:**
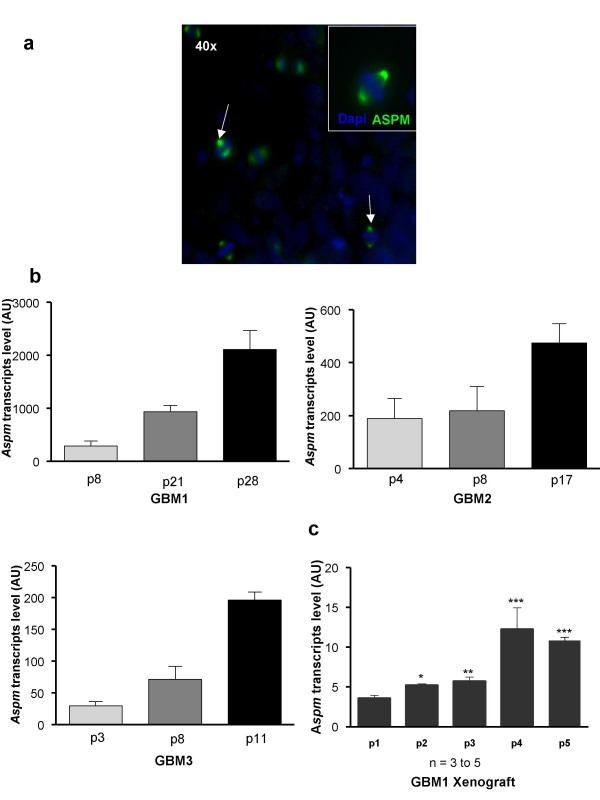
**ASPM expression in gliomaspheres increases with successive passages *in vitro *and *in vivo***. **(a) **Metaphase staining of ASPM protein at both poles of the spindle. ASPM protein (green) is detected in gliomaspheres (arrow). Nuclei are stained with DAPI (blue); **(b) **ASPM expression increases with successive passages (p) in gliomaspheres issued from GBM 1, 2 and 3. Passages were performed *in vitro *every 8 to 12 days; **(c) **GBM1 cells were subcutaneously engrafted into nude mice and *Aspm *expression was measured over four passages. *Aspm *expression increased progressively in xenograft tumor (mean +/- SEM; n = 3 to 5 mice for each point. *In vivo *passages were performed every 8-16 weeks.

We generated three gliomaspheres cultures, GBM1, GBM2, GBM3, all characterized by EGFR amplification and 10q loss on CGH-array analysis; this genetic profile remained stable throughout the passages (as shown in additional file 2, Figure S2 for GBM1). We also verified the capacity of gliomaspheres to differentiate in the presence of fetal bovine serum and to express multilineage markers (additional file 2, Figure S2). After dissociation of gliomaspheres, ASPM was detected during mitosis and located at the spindle poles of dividing cells (Figure 2a). ASPM transcript expression significantly increased throughout the passages for all gliomaspheres studied (Figure 2b). For example, in GBM1 gliomaspheres, we observed a 6.5-fold increase in ASPM mRNA level between passages 8 and 28.

After four passages and after checking genomic stability via CGH-a (not shown), 500,000 dissociated cells from GBM1 gliomaspheres were injected subcutaneously into immunodeficient nude mice. ASPM mRNA, measured by RT-PCR, increased progressively with successive tumor passages (Figure 2c).

### 4. ASPM silencing inhibits cell proliferation/gliomasphere formation and promotes cell death

**Figure 3:**
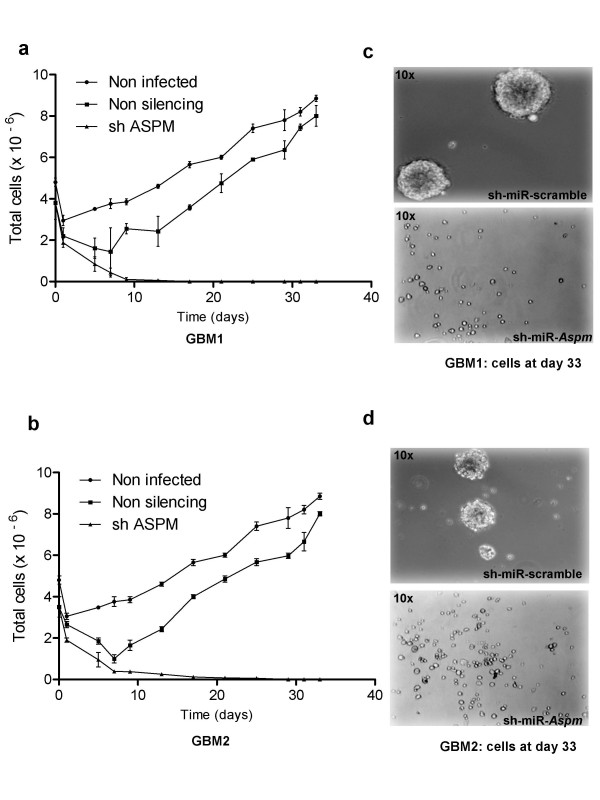
**ASPM knockdown in glioblastoma gliomaspheres**. Cell kinetics of GBM1 **(a) **and GBM2 **(b) **gliomaspheres. The data were generated from three independent experiments. Gliomaspheres treated with sh-miR-*Aspm *do not grow as compared to non-transduced gliomaspheres and gliomaspheres transduced with non-silencing sh-miR-RNA, which start growing again after a transient drop; **(c, d) **at day 33, there is extensive cell death in cell populations of GBM1 and GBM2 expressing the ASPM-silencing sh-miR-RNA as compared to the cells expressing a non-targeted sh-miR-RNA.

**Figure 4:**
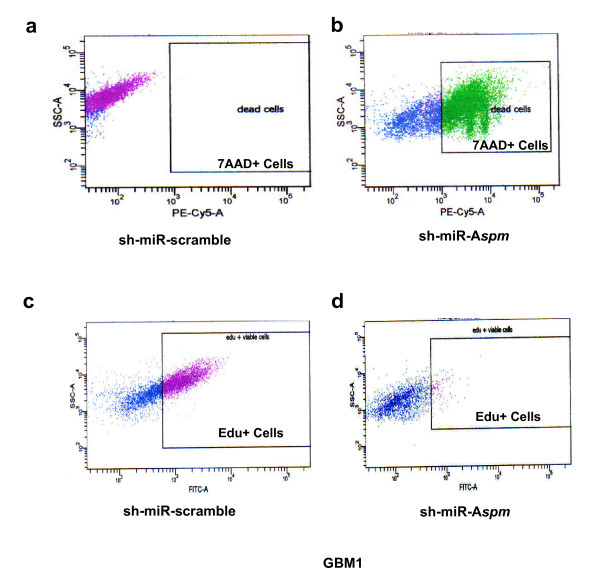
**Cell death and proliferation inhibition induced by ASPM silencing, evaluated by 7-Actinomycin D (7-AAD) labeling and 5- Ethyl -2'- Deoxyuridine (EdU) incorporation**. Compared to control **(a), **transduction of GBM1 with sh-miR-*Aspm *results in massive cell death **(b) **and a marked decrease in proliferation of surviving cells, with only 25% of the viable cells being EdU+ **(d)**, compared to 64% for the controls **(c)**.

To determine the role of ASPM, we infected gliomaspheres with lentivirus expressing a sh-miR-RNA that specifically target *Aspm*. In GBM1, GBM2 and GBM3 gliomasphere models, ~80% of cells were successfully transduced with the sh-miR-RNA specific to *Aspm*. In transduced cells, ASPM expression measured by RT-PCR was dramatically reduced to less than 5% in GBM2 and less than 0.02% in GBM1 and GBM3 (additional file 3, Figure S3). We then analyzed cell kinetics in three different groups: non-transduced cells, cells transduced with the non-targeted sh-miR-RNA, and cells transduced with sh-miR-*Aspm*. After day 5, cells transduced with the non-silencing sh-miR-RNA proliferated again while the sh-miR-*Aspm *cells died (Figure 3a-b). The morphology of the cells is shown at day 33 in Figure 3c and 3d. We then quantified the effects of *Aspm *silencing on proliferation (assessed by 5-Ethyl -2'-Deoxyuridine (EdU) incorporation) and cell death (assessed by 7-AAD^+ ^labeling). We noted an accumulation of dead cells in sh-miR-*Aspm *populations (80% of 7-AAD^+^GBM1, as shown in Figure 4b, and 85% of 7-AAD^+ ^GBM2) compared to control (Figure 4a). In addition to cell death, ASPM inhibition resulted in a decrease in proliferation: in sh-miR-*Aspm *GBM1 gliomaspheres, only 25% of the viable cells (i.e., 5% of total cells) were EdU+ cells (Figure 4d), compared to 64% in the non-silenced cell population (Figure 4c). For GBM2, the results were similar: 4% of 15% of viable cells were EDU+ in sh-miR-*Aspm *GBM2 gliomaspheres *vs*. 81% in control GBM2 (data not shown).

### 5. ASPM silencing inhibited gliomasphere formation of GBM cancer stems cells

In order to evaluate more specifically the impact of *Aspm *silencing on GBM cancer stem cells, we conducted a secondary gliomasphere-forming assay in GBM1. Seven days after silencing, GFP^+ ^cells were sorted and re-incubated at 1,000 cell/10 mL. We observed a dramatic decrease in cell number in GBM1 sh-miR-*Aspm *compared to the non-silenced control (1000 cells *vs*. 2.8.10^5 ^cells at day 9 and 500 cells *vs*. 3.7.10^6 ^cells at day 15). At D15, no gliomaspheres were observed (*vs*. 91 gliomaspheres in the control), indicating that the knockdown of *Aspm *completely abrogated the development of secondary gliomaspheres.

## Discussion

This study indicates that ASPM is tightly associated with the malignant progression of gliomas and that its knockdown produces extensive death of glioma cells *in vitro*.

As previously described [7], we found that ASPM expression was strongly correlated with the grade of gliomas. In recurrent tumors, ASPM expression increased not only in patients who progressed to a higher grade, but also in recurrent glioblastomas, suggesting that a progressive increase in ASPM mRNA expression is a marker for the inexorable malignant progression and aggressiveness of gliomas, continuing even when the tumors have already reached their most anaplastic histological stage (Grade IV). Experimental evidence supports this finding since we found a progressive increase in ASPM mRNA expression in serial passages of gliomaspheres *in vitro *and in mouse glioma xenografts *in vivo*. To explain such a tight correlation between ASPM expression and the malignant progression of tumors, an attractive hypothesis is that symmetric division of CSC-expressing ASPM leads to progressive proliferative expansion of this undifferentiated compartment. Although we found that ASPM was also expressed at the protein level in all glioma grades and overexpressed in Grade IV gliomas, it is worth noting that the results were less striking. This finding has been previously noted and led to the suggestion that ASPM could be in part regulated at the translational level [7]. However, the dramatic inhibition of gliomasphere proliferation obtained with shRNA-mediated ASPM knockdown highlights the potential importance of this gene in malignant gliomas. Sphere formation is a key behavior of neural and brain tumor stem cells and is used to test stem cell capacity for self-renewal. A previous study also showed that siRNA-mediated inhibition of ASPM resulted in cell proliferation inhibition, thought to be mediated by a G1-phase cell cycle arrest [6]. The effect that we observed here by stable transduction of shRNA expression in CSC-enriched cells appears even more drastic, possibly because of a more efficient and longer lasting inhibition of *ASPM*. Whereas siRNA transfection results in a transient effect, lentiviral-mediated expression of shRNA allows the transgene to integrate into the genome of the host cell, resulting in long-term expression. Production of secondary spheres was also inhibited after ASPM knockdown, a finding compatible with a sustained inhibition of CSC proliferation. Interestingly, stable inhibition of ASPM not only prevented further growth but also produced rapid and extensive cellular apoptotic death in our model. This finding may indicate that, following ASPM inhibition, CSC underwent active maturation and differentiation but could not survive in our experimental setting (serum-free medium without appropriate growth factors). However, experiments by Horvath et *al*. conducted in a similar serum-free medium resulted in growth inhibition and not massive cell death. On the other hand, the function of *ASPM *in neoplastic cells, particularly gliomas, has not been completely elucidated. In particular, the links between ASPM and key molecular alterations of malignant gliomas, such as EGFR amplification or mutation (EGFRvIII) (an almost constant feature in gliomaspheres - 80% in our experience), remain to be investigated. Nevertheless, some data suggest that these alterations are closely related. ASPM mRNA expression was increased in an EGFRvIII-expressing U87 glioblatoma cell line as compared to an isogenic U87 line, while ASPM expression was inhibited following the addition of erlotinib (an EGFR tyrosine kinase inhibitor) to the EGFRvIII-expressing U87 line. This observation led Horvath et *al *to suggest that ASPM (and genes from the same module) is involved downstream of the mutant EGFR. Thus, inhibition of ASPM could prove lethal by severely hampering one of the key signaling pathways of glioblastomas.

Because ASPM is absent or expressed at very low levels in normal brain, the considerable differential expression levels between GBM and normal parenchyma and the extreme *in vitro *sensitivity of tumor cells to ASPM knockdown support continued research to specifically target ASPM from a therapeutic perspective, focusing on *in vivo *studies.

## Competing interests

The authors declare that they have no competing interests.

## Authors' contributions

SNNB, CC and YM carried out most of the in vitro work. RV and PR produced the lentivirus. SNNB. AR and BB performed the immunohistochemistry experiments. AI performed the CGH-array experiments. PL and ML did the in vivo experiments. SEH contributed to the in vitro experiments. MS conceived the study. JYD and MS wrote the manuscript. All authors read and approved the final manuscript.

## Supplementary Material

Additional file 1 **Figure S1 - ASPM protein expression**. ASPM western blot analysis of grade II, III and IV gliomas (n = 4 in each group). Relative protein expression is presented as the ratio of density value for ASPM over GAPDH signal (mean +/- SEM). Click here for file

Additional file 2 **Figure S2 - Tumor spheroid characterization**. Genomic stability was examined with CGHa analysis (left = DNA profile from initial tumor; right = DNA profile from gliomasphere at passage p28). The chromosomes are indicated on the x axis and copy number is on y axis. Yellow indicates the normal genomic copy number, while green indicates a loss and red indicates a gain in copy number. As shown here, GBM1 carries EGFR amplification and loss of chromosome 10q. After CSC isolation in stem cell-permissive medium, gliomaspheres were differentiated in presence of fetal bovine serum and stained by Sox2 and O4 for stemness markers and by GFAP and Tuji1 to characterize the potential of multi lineage differentiation of GBM1. Click here for file

Additional file 3 **Figure S3 - Knock-down of Aspm gene**. Tumor spheroids (GBM1, GBM2, GBM3) were transfected either with non silencing (scrambled ShRNA) or with ASPM ShRNA expressing lentivirus, resulting in a dramatic drop of ASPM RNA in GBM1, GBM3 and to a lesser extent GBM2. Click here for file
