# Correction: ASPM-associated stem cell proliferation is involved in malignant progression of gliomas and constitutes an attractive therapeutic target

**DOI:** 10.1186/1475-2867-11-10

**Published:** 2011-04-15

**Authors:** Sandra-Nadia Ngwabyt Bikeye, Carole Colin, Yannick Marie, Raphaël Vampouille, Philippe Ravassard, Audrey Rousseau, Blandine Boisselier, Ahmed Idbaih, Charles Félix Calvo, Pascal Leuraud, Myriam Lassalle, Soufiane El Hallani, Jean-Yves Delattre, Marc Sanson

**Affiliations:** 1 UMR975 INSERM-UPMC, GH Pitié- Salpêtrière, Paris, France; 2 UMR911-CRO2, Faculté de Médecine Timone, Université de la Méditerranée, Marseille, France; 3 Service de Neurologie Mazarin, GH Pitié- Salpêtrière, APHP, Paris, France; 4 Biotechnology & Biotherapy laboratory, Centre de Recherche de l'Institut du Cerveau et de la Moelle, CNRS UMR9225, INSERM UMRS 975 Université Pierre et Marie Curie, Hôpital de la Salpêtrière, Paris, France; 5 Service de Neuropathologie Raymond Escourolle, GH Pitié- Salpêtrière, Paris, France; 6 XenTech, Evry, France

## Correction

In the original article [1], the additional file two: figure S.Two (additional file 1 here) contained two identical graphs. We present here the corrected figure S2 and the corresponding legend:

## Supplementary Material

Additional file 1 **Tumor spheroid characterization**. Genomic stability was examined with CGHa analysis (left = DNA profile from the initial tumor; right = DNA profile from gliomasphere at passage p28). The chromosomes are indicated on the × axis and copy number is on y axis. Yellow indicates the normal genomic copy number, while green indicates a loss and red indicates a gain in copy number. Although some differences are observed, the comparison of both profiles shows that overall genomic profile is quite well preserved (except for an amplicon on chromosome 8 present only in the gliomasphere). Click here for file
