# Supplementary figures and images for: ASPM-associated stem cell proliferation is involved in malignant progression of gliomas and constitutes an attractive therapeutic target
Source: Cancer Cell Int. 2010 Jan 11;10:1. doi: 10.1186/1475-2867-10-1 (PMC2817685; doi:10.1186/1475-2867-10-1)

## Slide 1
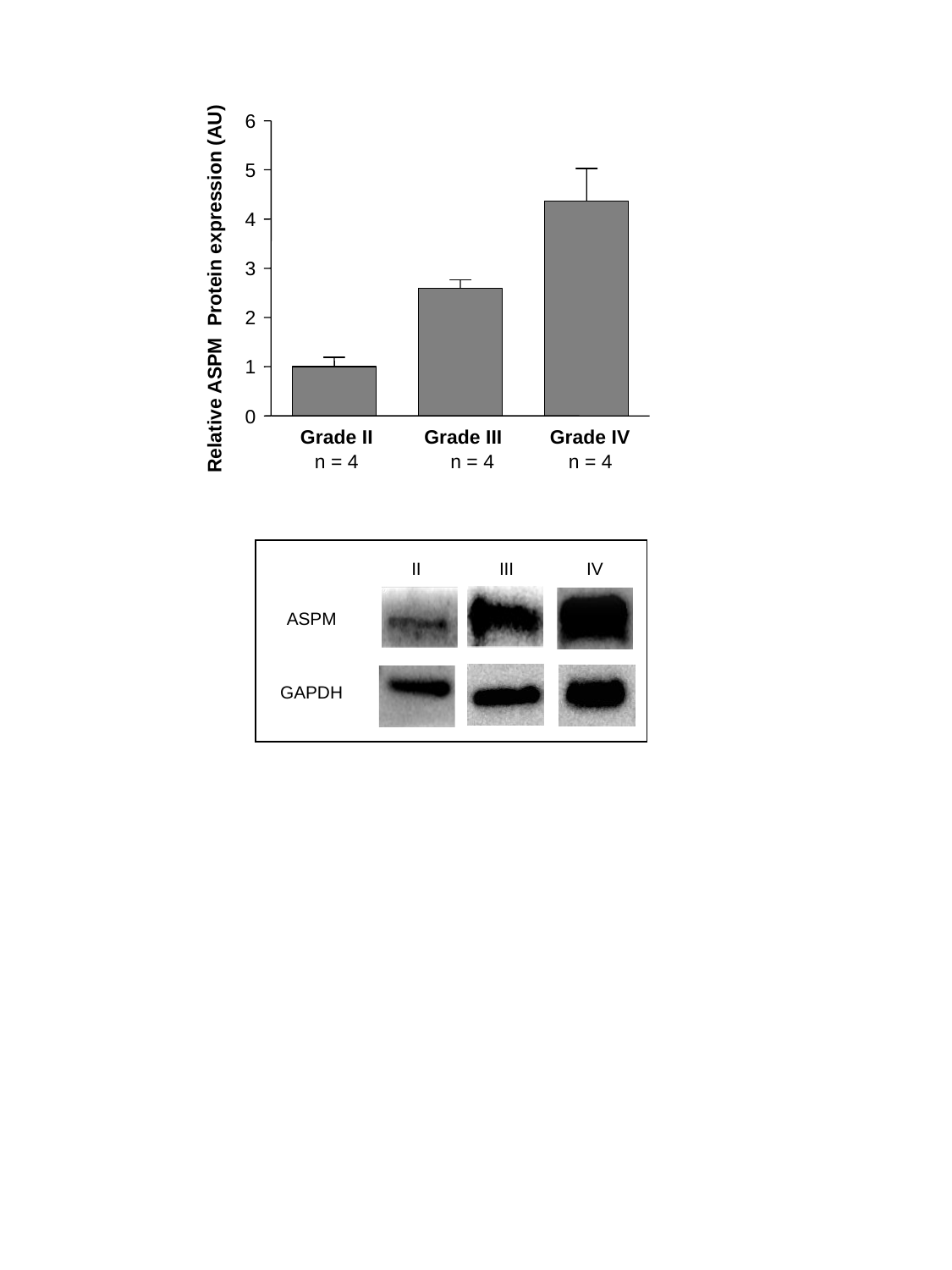

6
5
4
3
Relative ASPM Protein expression (AU)
2
1
0
Grade II
Grade III
Grade IV
n = 4
n = 4
n = 4
II
III
IV
ASPM
GAPDH

Supplement: Additional file 1 — Figure S1 - ASPM protein expression. ASPM western blot analysis of grade II, III and IV gliomas (n = 4 in each group). Relative protein expression is presented as the ratio of density value for ASPM over GAPDH signal (mean +/- SEM). [file 1475-2867-10-1-S1.PPT]

## Slide 1
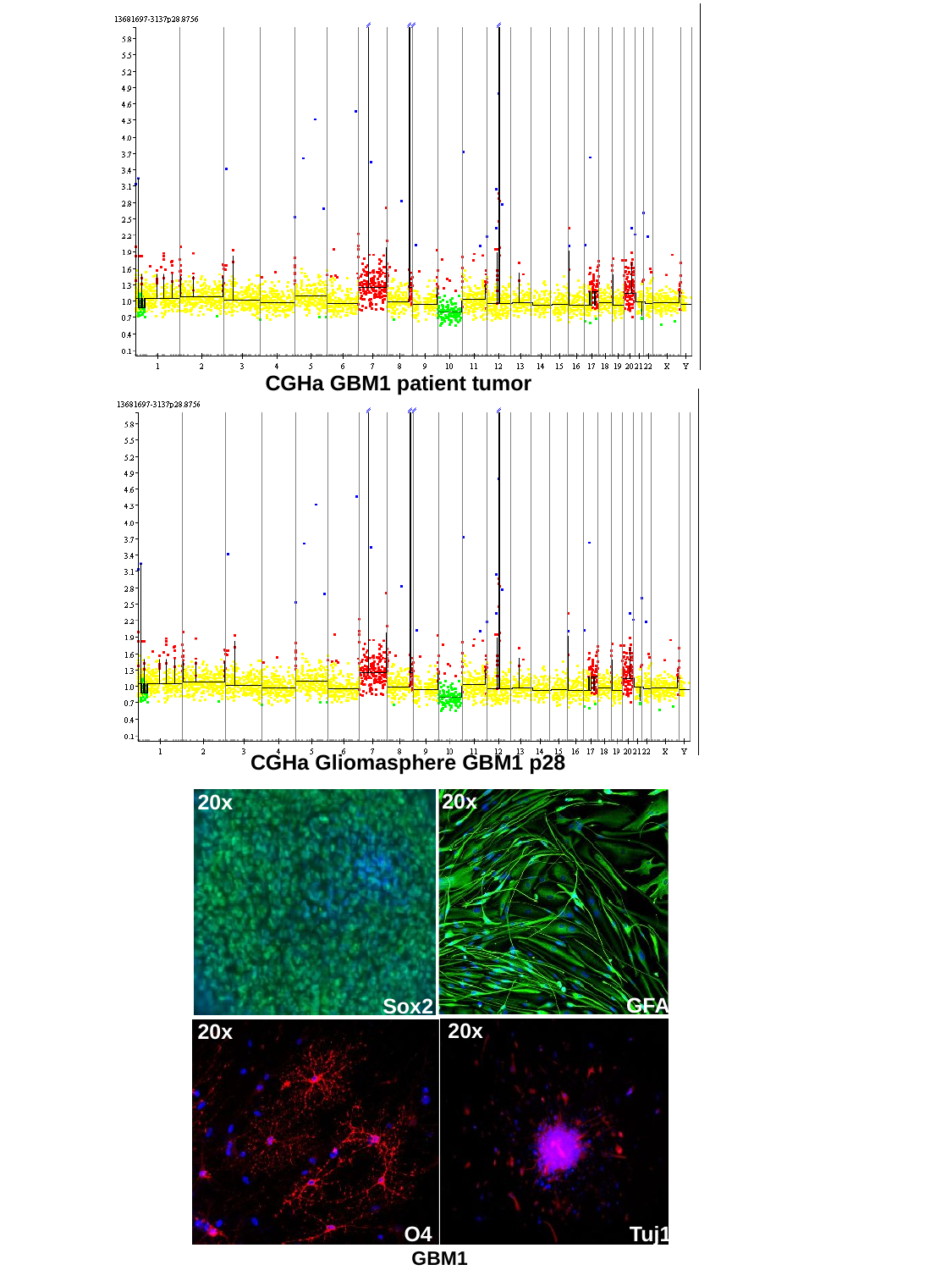

CGHa GBM1 patient tumor
CGHa Gliomasphere GBM1 p28
20x
20x
GFAP
Sox2
20x
20x
O4
Tuj1
GBM1

Supplement: Additional file 2 — Figure S2 - Tumor spheroid characterization. Genomic stability was examined with CGHa analysis (left = DNA profile from initial tumor; right = DNA profile from gliomasphere at passage p28). The chromosomes are indicated on the x axis and copy number is on y axis. Yellow indicates the normal genomic copy number, while green indicates a loss and red indicates a gain in copy number. As shown here, GBM1 carries EGFR amplification and loss of chromosome 10q. After CSC isolation in stem cell-permissive medium, gliomaspheres were differentiated in presence of fetal bovine serum and stained by Sox2 and O4 for stemness markers and by GFAP and Tuji1 to characterize the potential of multi lineage differentiation of GBM1. [file 1475-2867-10-1-S2.PPT]
