# Supplementary material for: ASPM-associated stem cell proliferation is involved in malignant progression of gliomas and constitutes an attractive therapeutic target
Source: Cancer Cell Int. 2010 Jan 11;10:1. doi: 10.1186/1475-2867-10-1 (PMC2817685; doi:10.1186/1475-2867-10-1)
Supplement: Additional file 3 — Figure S3 - Knock-down of Aspm gene. Tumor spheroids (GBM1, GBM2, GBM3) were transfected either with non silencing (scrambled ShRNA) or with ASPM ShRNA expressing lentivirus, resulting in a dramatic drop of ASPM RNA in GBM1, GBM3 and to a lesser extent GBM2. [file 1475-2867-10-1-S3.PPT]

## Slide 1
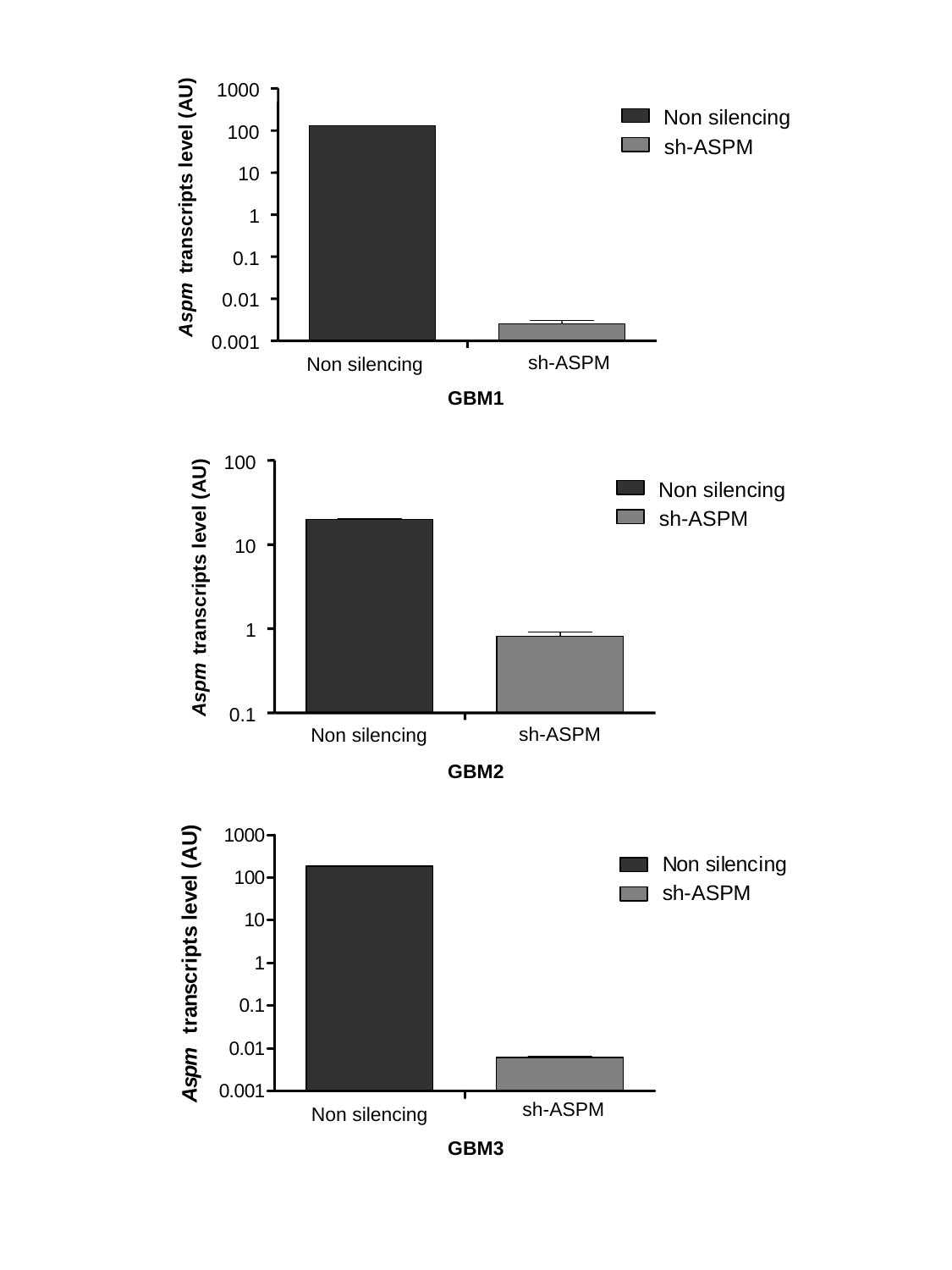

transcripts level (AU)
Aspm
1000
Non silencing
100
sh-ASPM
10
1
0.1
0.01
0.001
sh-ASPM
Non silencing
GBM1
100
 transcripts level (AU)
Aspm
Non silencing
sh-ASPM
10
1
0.1
sh-ASPM
Non silencing
GBM2
sh-ASPM
Non silencing
GBM3
