# Supplementary figures and images for: Correction: ASPM-associated stem cell proliferation is involved in malignant progression of gliomas and constitutes an attractive therapeutic target
Source: Cancer Cell Int. 2011 Apr 15;11:10. doi: 10.1186/1475-2867-11-10 (PMC3083327; doi:10.1186/1475-2867-11-10)

## Slide 1
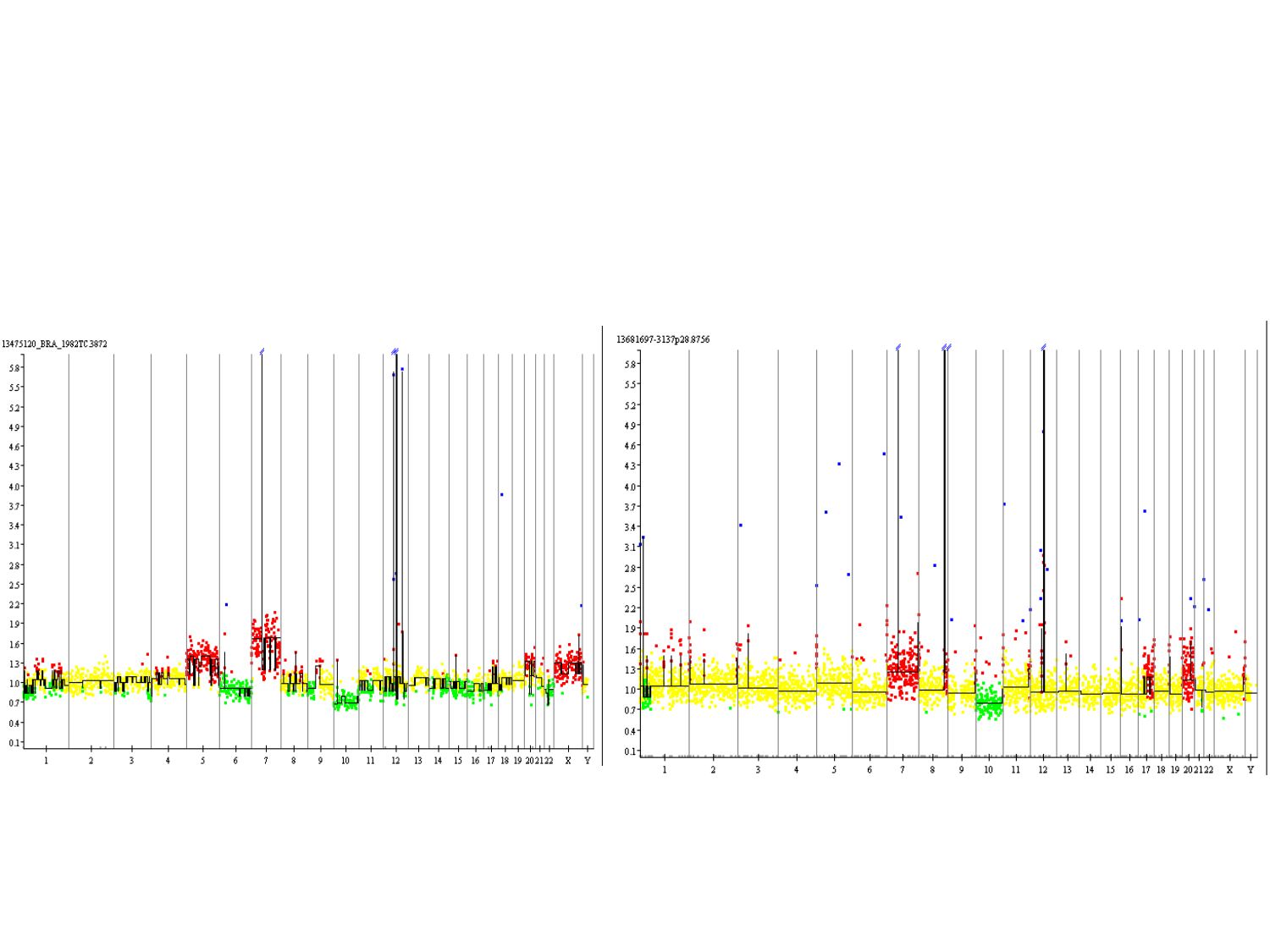

Supplement: Additional file 1 — Tumor spheroid characterization. Genomic stability was examined with CGHa analysis (left = DNA profile from the initial tumor; right = DNA profile from gliomasphere at passage p28). The chromosomes are indicated on the × axis and copy number is on y axis. Yellow indicates the normal genomic copy number, while green indicates a loss and red indicates a gain in copy number. Although some differences are observed, the comparison of both profiles shows that overall genomic profile is quite well preserved (except for an amplicon on chromosome 8 present only in the gliomasphere). [file 1475-2867-11-10-S1.PPT]
